# Imaging conformations of *holo*- and *apo*-transferrin on the single-molecule level by low-energy electron holography

**DOI:** 10.1038/s41598-023-37116-x

**Published:** 2023-06-23

**Authors:** Hannah Ochner, Sven Szilagyi, Moritz Edte, Tim K. Esser, Stephan Rauschenbach, Luigi Malavolti, Klaus Kern

**Affiliations:** 1 Max Planck Institute for Solid State Research, Heisenbergstr. 1, 70569 Stuttgart, Germany; 2 Department of Chemistry, University of Oxford, 12 Mansfield Road, Oxford, OX1 3TA UK; 3 Institut de Physique, École Polytechnique Fédérale de Lausanne, 1015 Lausanne, Switzerland; 4 Present Address: Thermo Fisher Scientific, 1 Boundary Park, Hemel Hempstead, HP2 7GE UK

**Keywords:** Microscopy, Imaging techniques

## Abstract

Conformational changes play a key role in the biological function of many proteins, thereby sustaining a multitude of processes essential to life. Thus, the imaging of the conformational space of proteins exhibiting such conformational changes is of great interest. Low-energy electron holography (LEEH) in combination with native electrospray ion beam deposition (ES-IBD) has recently been demonstrated to be capable of exploring the conformational space of conformationally highly variable proteins on the single-molecule level. While the previously studied conformations were induced by changes in environment, it is of relevance to assess the performance of this imaging method when applied to protein conformations inherently tied to a function-related conformational change. We show that LEEH imaging can distinguish different conformations of transferrin, the major iron transport protein in many organisms, by resolving a nanometer-scale cleft in the structure of the iron-free molecule (*apo*-transferrin) resulting from the conformational change associated with the iron binding/release process. This, along with a statistical analysis of the data, which evidences a degree of flexibility of the molecules, indicates that LEEH is a viable technique for imaging function-related conformational changes in individual proteins.

## Introduction

Low-energy electron holography (LEEH)^1^ has been proven capable of imaging proteins at the single-molecule level with subnanometer resolution^2–4^. In particular, LEEH in combination with native electrospray ion beam deposition (ES-IBD) can provide information on a protein’s conformational space and thus allows the investigation of biomolecules in different environmentally-induced conformations^3^. This is of particular relevance for the study of proteins exhibiting a high degree of conformational variability, which can remain elusive when studied by ensemble-averaging techniques such as electron cryomicroscopy (cryoEM) and X-ray crystallography^5,6^. Conformational changes in proteins, however, cannot only be traced to alterations in the molecules’ environment, but often are tied to biological processes related to the protein’s function. Hence, it is of interest to analyze the performance of LEEH, which is a relatively novel approach to protein imaging and thus still offers a range of largely unexplored possibilities, regarding the imaging of a protein in different conformations that are known to be inherently tied to its biological function.

**Figure 1:**
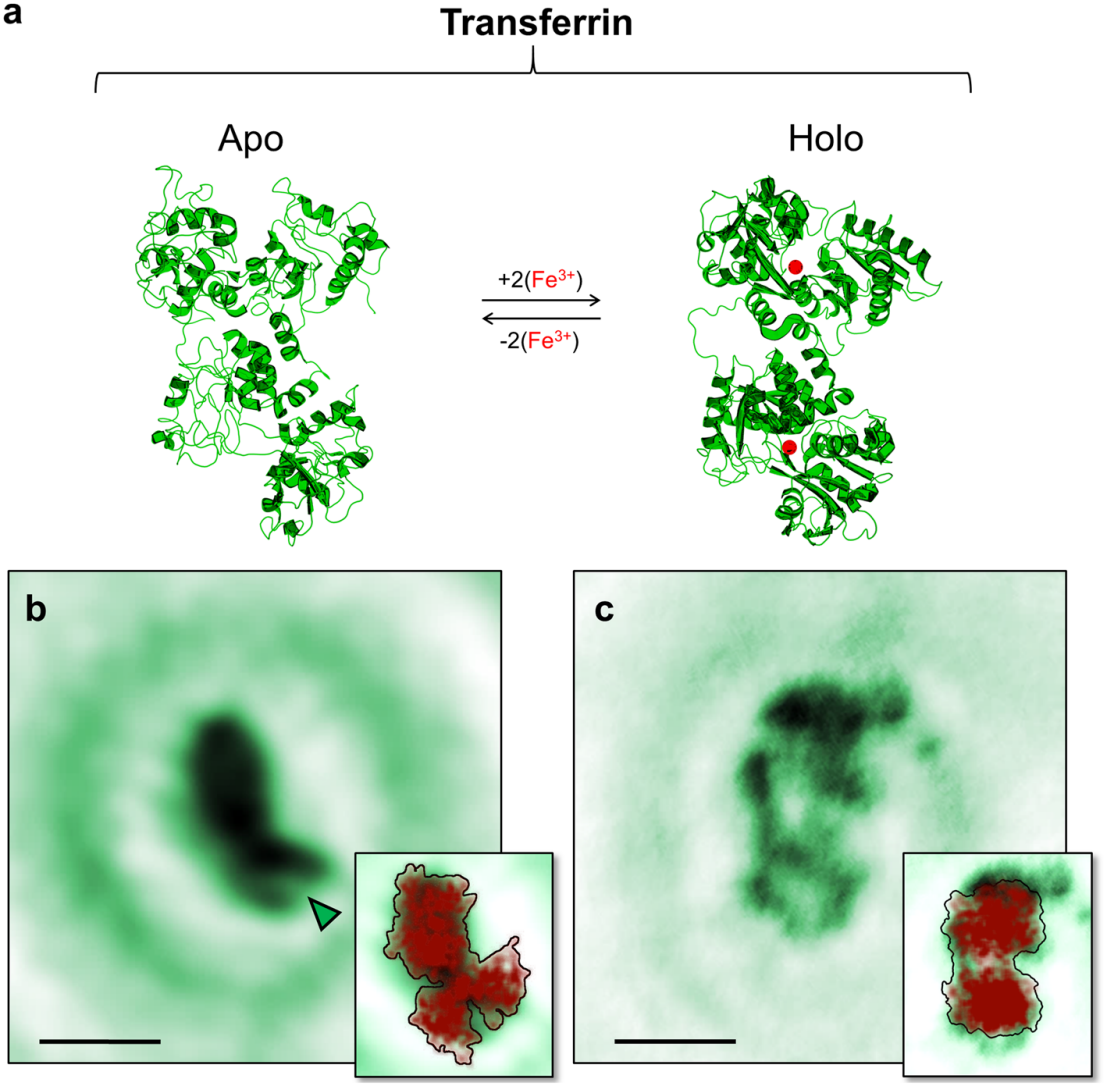
Imaging *apo*- and *holo*-transferrin conformations. (**a**) PDB models of *apo*- (PDB: 1RYX^15^) and *holo*-transferrin (PDB: 1JNF^16^). Transferrin molecules switch between the *apo*- and *holo*-forms by the binding and releasing of iron ions (indicated as red spheres in the *holo*-transferrin model). The associated conformational change manifests by the appearance of clefts in the lobes of *apo*-transferrin molecules.)** b**) One-step amplitude reconstruction of a hologram of an individual *apo*-transferrin molecule experimentally acquired by a LEEH measurement. On one of the lobes, the cleft defining the open lobe conformations is clearly visible, as indicated by the green arrow. Scale bar: 5 nm. (**c**) One-step amplitude reconstruction of a hologram of an individual *holo*-transferrin molecule experimentally acquired by a LEEH measurement. Scale bar: 5 nm. Insets in (**b**) and (**c**) show the experimental image superimposed by a projection of the PDB model in an orientation corresponding to that of the imaged molecule.

A class of proteins showing such a function-related conformational change are transferrins. Transferrins are the major iron transport proteins in many organisms and are thus of critical importance for maintaining iron homeostasis^7–9^. To fulfil their biological role, transferrins can bind and release iron ions via a mechanism associated with a well-known conformational change^8–16^. Structurally, transferrins consist of a single peptide chain formed into two homologous lobes, the N-terminal lobe and the C-terminal lobe. Each of these lobes features a metal-binding site, thus a transferrin molecule can bind and transport up to two ferric ions simultaneously. Depending on whether iron is bound or not, transferrin can exist in two main conformations: without bound iron ions, the lobes exhibit a cleft (*open* conformation), whereas the binding of iron ions results in a compact lobe structure (*closed* conformation)^8–16^ (Fig. 1a). In the following, we will use the terms *open* and *closed* conformation to specify the conformational state of the lobes and *holo*- and *apo*-transferrin to distinguish between diferric and iron-free molecules. While the presence (absence) of the clefts upon releasing (binding) of the iron ions is the clearest structural alteration, the conformational change associated with the transition between the *holo*- and *apo*-transferrin forms also affects the relative orientation of the lobes within the molecules via a readjustment of the connecting region between the two lobes^10,14^. Since transferrins have been extensively studied both experimentally and computationally^8–14^, these structural changes are well-understood and thus set a benchmark for assessing the performance of our LEEH investigation regarding the mapping of a protein’s conformational space defined by a biologically relevant conformational change.

Here, by separately depositing *holo*- and *apo*-transferrin using native Electrospray Ion Beam Deposition (ES-IBD)^17^ on a single layer graphene (SLG) substrate and subsequently investigating the observed conformational space with LEEH, we show that LEEH imaging can map the *open* and *closed* lobe conformations associated with the *holo*- and *apo*-forms of the molecules. Multiple molecular shapes are observed in the LEEH images of both types of molecules, consistent with different adsorption orientations with respect to the graphene surface. The resulting variety of views is particularly significant since not all possible orientations allow the identification of the exact lobe conformation (*open* or *closed*). While in the case of *holo*-transferrin, all the imaged molecules show lobes in a *closed* conformation, for *apo*-transferrin the different molecular orientations lead to a more complex scenario in which clear clefts are only visible on a subset of the molecules. Our statistical analysis shows that the amount of *open* lobe conformations observed in the LEEH experiments is in agreement with the percentage of clefts expected to be observable from a set of random orientations created from projections of the PDB model^15^ of an *apo*-transferrin molecule. Furthermore, the evaluation of the observed molecular size distribution reveals that the sizes of the individual lobes match those expected from the models, while the overall length of the molecules is on average slightly longer than expected, which indicates a degree of flexibility of the connecting region. These findings directly demonstrate LEEH’s ability to image conformations associated with small, function-related conformational changes in individual proteins.

## Results

Separate samples of human *apo*- and *holo*-transferrin molecules were created by bringing the respective molecules from pure solutions into the gas phase via native electrospray ionization, mass-selecting to avoid contamination (Supplementary Fig. 1), and subsequently soft-landing (landing energy < 5 eV per charge) the molecules on single layer graphene substrates at room temperature. The resulting *apo*- and *holo*-transferrin samples were imaged in our LEEH microscope (see Methods section), recording holograms of individual proteins. A one-step numerical reconstruction algorithm^2,3,18^ (see Methods section) was employed to retrieve a real-space image from the experimentally acquired hologram, yielding a 2D projection of the molecular shape and size. For details on the above steps, see Methods section.

In general, the ES-IBD sample preparation process will generate proteins in a variety of different orientations with respect to the graphene surface^3^. In the case of transferrin, this results in a range of orientations from molecules showing both lobes (Fig. 1b and c, approximately 40-65% of the total amount of observed molecules on a sample) to molecules exhibiting a compact structure with one of the lobes eclipsed by the other. The resulting diversity in orientation on the single layer graphene surface, and thus in overall molecular shape, can be mapped by LEEH imaging (see Supplementary Fig. 2). For each of the observed shapes, a matching projection from the crystallographic model can be found, indicating that both *apo*- and *holo*-transferrin molecules remain close to the native structure during ES-IBD sample preparation and LEEH imaging. Since the observation of molecules clearly exhibiting both lobes is beneficial for distingusihing *open* and *closed* lobe conformations, we initially focus our discussion on these structures.

Fig. 1b and c show an example of an *apo*- and a *holo*-transferrin molecule with two clearly visible lobes, respectively. While the *holo*-transferrin molecule (Fig. 1c) presents the two lobes in a similar, compact shape corresponding to a closed lobe conformation, the *apo*-transferrin molecule exhibits a clearly distinguishable cleft in one lobe (Fig. 1b). This difference between *holo*- and *apo*-transferrin is validated by comparing a large number of reconstructed images obtained by LEEH imaging: lobes that are unequivocally associated with open conformations, i. e. lobes with a clearly visible cleft, are only observed in the samples prepared from *apo*-transferrin molecules. The clefts obtained from LEEH imaging have an average width at maximum opening of $2.1\pm 0.5\,\textrm{nm}$ and an average length of $1.9\pm 0.5\,\textrm{nm}$, which matches the dimensions of the cleft observable in X-ray crystallography structures of *apo*-transferrin (e.g. 1RYX^15^). Next to the experimental data, a projection of the crystallographic model (PDB: 1JNF^16^ (*holo*), PDB: 1RYX^15^ (*apo*)) in an orientation corresponding to that of the experimentally observed molecule is displayed in the insets of Fig. 1b and c. The reconstructed images closely resemble the projections of the model both in overall shape and size and on the level of the individual lobes for both molecules. While the overall molecular dimensions are almost identical in the case of *apo*-transferrin, a slight discrepancy in the molecule’s length is apparent for *holo*-transferrin. This deviation does not affect the identification of *open* and *closed* lobe conformations and will be further discussed in the context of the statistical analysis presented in Fig. 3. It is relevant to note here that the projected model reproduces the cleft defining the *open* lobe conformation of the *apo*-transferrin molecule while simultaneously accounting for the bulky appearance of the other lobe, in which the cleft is not visible. Indeed, because of the clefts’ relative locations, defined by the orientation of the two lobes with respect to one another^10^, only one cleft is visible while the cleft on the other lobe remains obscured. Hence, the projection of one of the lobes in an *open* conformation resembles that of a *closed* lobe. This similarity is evident both on the level of the crystallographic structures of *apo*- and *holo*-transferrin as well as when comparing the images reconstructed from experimental holograms, in which *closed * lobe conformations, as exhibited by *holo*-transferrin molecules, and eclipsed-cleft *open* lobe configurations in *apo*-transferrin molecules are virtually indistinguishable (Fig. 1b and c).

**Figure 2:**
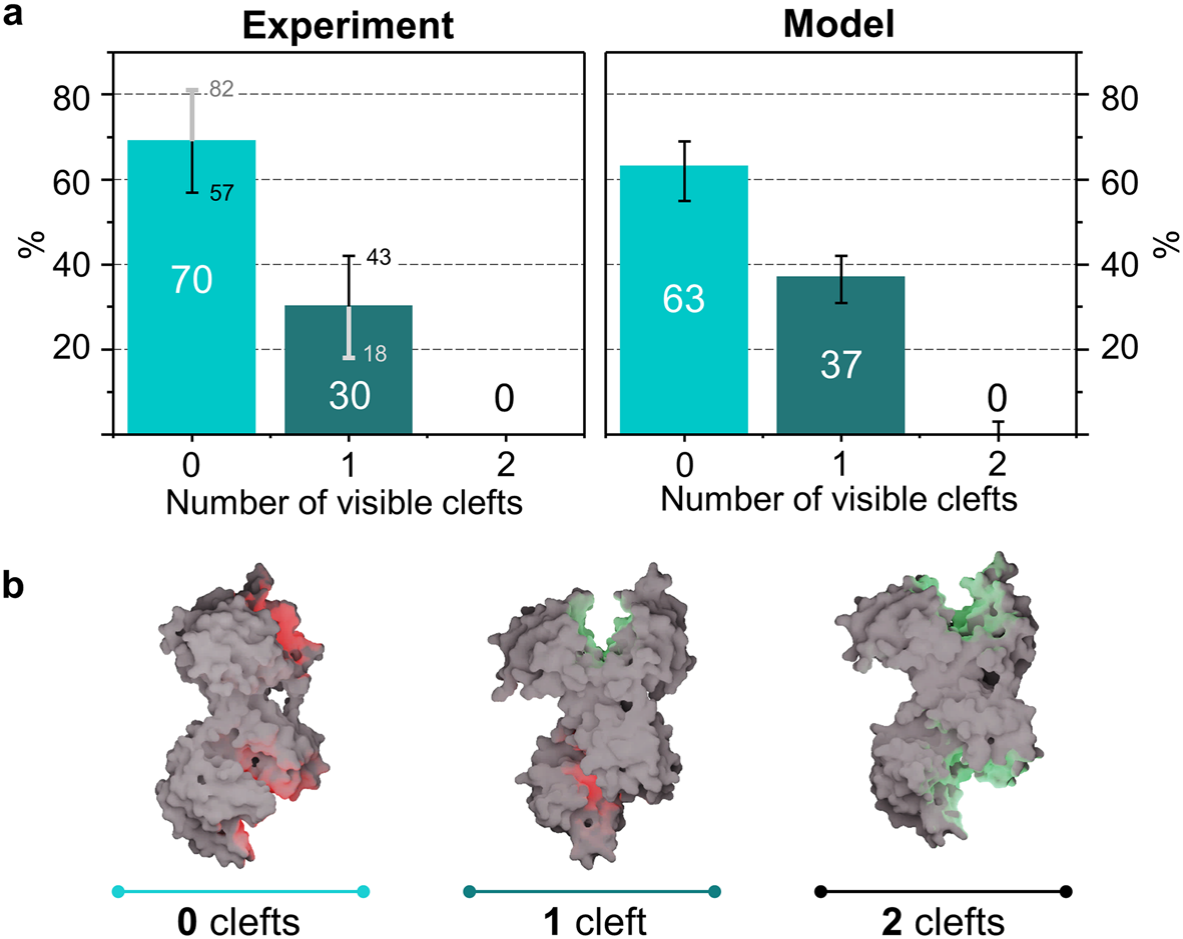
Visibility of the cleft in *apo*-transferrin depending on surface orientation. (**a**) Number of visible clefts per molecule in the LEEH experiment (left) and in the distribution obtained from a set of random projections generated from the PDB model (right). Experiment (on average 30% of the observed molecules feature one visible cleft) and model (one visible cleft observable on 37% of the molecules) are in good agreement. The error bar on the experimental data is based on the different counts of molecules with clearly visible clefts only (18%) and of those including ambiguous cases (43%). For the derivation of the error bars on the model data, see Supplementary Information. (**b**) Illustration of orientations featuring 0, 1 and 2 visible clefts. Visible clefts are indicated in green, obscured clefts in red.

Despite the fact that both lobes of an *apo*-transferrin molecule are in an *open* conformation, our statistical analysis of the experimental data shows that none of the observed molecules features two lobes clearly presenting a cleft. Additionally, lobes exhibiting a clearly recognizable open conformation were only observed in 18% of the imaged *apo*-transferrin molecules, where a high-contrast cleft is defined by a drop in amplitude values to background level, as in the example in Fig. 1b. When taking into account more ambiguous lobe conformations that fit an open lobe conformation in overall shape, but do not feature a high-contrast cleft, which could be due to a reduction in contrast due to a degree of fuzziness at the boundary of the molecules, the percentage of observable *open* conformations in *apo*-transferrin molecules increases up to 43% (Fig. 2a).

On the vast majority of the investigated molecules, our imaging does not observe cleft openings. Since the diversity in surface orientation effectively hinders the unequivocal determination of the lobe conformations on part of the imaged molecules, we compared the number of *open* lobe conformations observed experimentally to the number of observable *open* conformations obtained from a set of 5000 random projections of the PDB model (1RYX^15^) of an *apo*-transferrin molecule (Fig. 2a). This is evaluated by calculating the visibility of the open cleft area in each of the random projections (see Supplementary Information, Supplementary Figs. 3 and 4). To define an unambiguously open cleft, we set a threshold area visibility of $12\,$Å$^2$, which roughly matches the area obtainable from the smallest feature so far observed by LEEH imaging (corresponding to approximately $5\,$ Å resolution).

The statistics obtained from the random projections of the model suggest that in 0% of the cases, both lobes should feature an open conformation (0% of the experimental cases), and in 37% of the cases, one lobe should be in an open conformation (30% on average in the experimental data, 18% of unambiguous cases, up to 43% if ambiguous cases are included). In the rest of the projections, which include compact conformations as well as extended conformations with no visible clefts, none of the lobes is classified as open. The statistical analysis reveals a high degree of similarity between the amount of expected *open* conformations from the random projections of the model and the percentage of experimentally observed *open* conformations. Small discrepancies might be due to the interaction with the graphene substrate, which may favour certain molecular orientations. The comparison between experiment and model thus suggests a correspondence between the *apo*-transferrin X-ray structure (1RYX^15^) and the on-surface *apo*-transferrin conformations measured by LEEH, confirming that the amount of experimentally observed clefts is reasonable for molecules whose structure closely resembles that of the crystallographic model.

**Figure 3:**
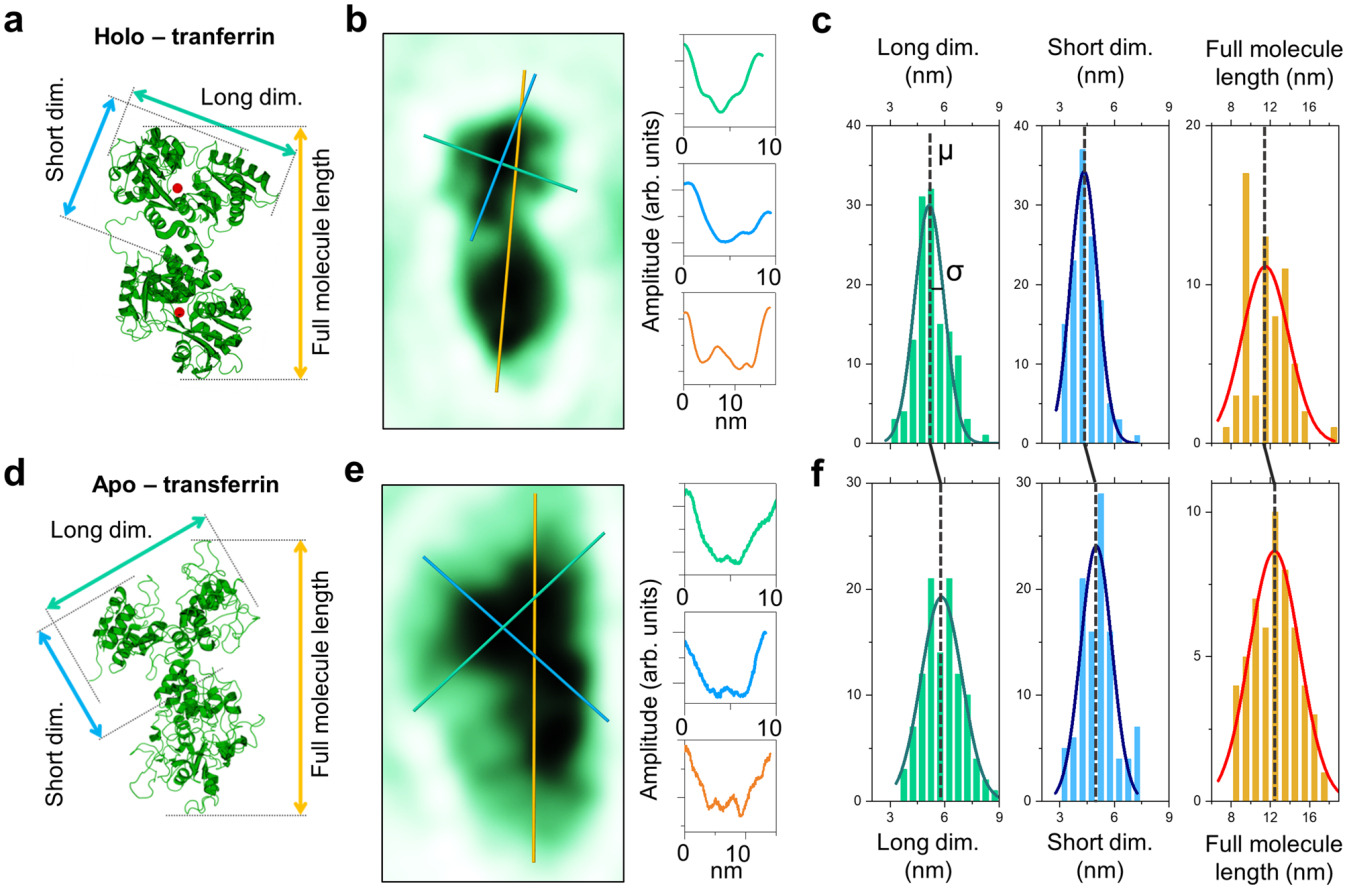
Size distribution of the lobe dimensions and the full molecule length. (**a**) Model of *holo*-transferrin indicating the different sizes (long lobe dimension, short lobe dimension, full molecule length) evaluated here. Since each molecule’s orientation determines whether the longer or the shorter lobe dimension is approximately aligned with the full-length axis, we use long and short dimension here rather than length and width. (**b**) LEEH amplitude reconstruction of an individual *holo*-transferrin molecule and cross sections for long lobe dimension, short lobe dimension and full molecule length as indicated by the green, blue and yellow lines. (**c**) Experimentally obtained size distributions of long lobe dimension, short lobe dimension, and full molecule length for *holo*-transferrin fitted with Gaussians. The mean values and standard deviations of the distributions are given in Table 1. (**d**)–(**f**) Corresponding data for *apo*-transferrin.

In addition, the observed molecular size distribution suggests a degree of flexibility of the connecting region in both *apo*- and *holo*-transferrin molecules. A statistical analysis of the lobe sizes (Fig. 3) shows that the size distribution of the individual lobes measured by LEEH matches the lobe sizes of the crystallographic model (long and short lobe dimensions of about 5.5 nm and 4 nm, respectively, as measured according to Fig. 3a and d). This confirms the preservation of the structural integrity of the molecules during the sample preparation process within the limit of the LEEH spatial resolution. When comparing the overall length of the molecules observed in extended two-lobe orientations to the full model length (maximum model length 9.5 nm for *holo*-transferrin and 10.5 nm for *apo*-transferrin), however, the experimentally measured structures (Fig. 3b and e) are on average slightly elongated, as demonstrated by the broad full-length distributions centred at 11.5 nm (*holo*) and 12.4 nm (*apo*), respectively (Fig. 3c and f).

This is further corroborated by comparing the size distribution of the individual lobes with the full-length distribution of the molecules (Table 1). For *holo*-transferrin, Gaussian fits of the lobe dimensions lead to peaks centred at 4.3 and 5.1 nm with standard deviation ($\sigma$) equal to 0.7 and 0.8 nm for short and long, respectively. A similar analysis on the full-length data leads to a Gaussian distribution centred at 11.5 nm with $\sigma =2.4\,\textrm{nm}$. In the case of *apo*-transferrin, the corresponding Gaussians are centred at 5.0 nm (short, $\sigma =0.8\,\textrm{nm}$), 5.8 nm (long, $\sigma =1.1\,\textrm{nm}$) and 12.4 nm (full length, $\sigma =2.5\,\textrm{nm}$). Not only is the mean ($\mu$) of the total molecular length larger than the sum of the means associated with the long lobe dimension for both *holo*- and *apo*-transferrin, but the standard deviation ($\sigma$) of the full-length distribution is larger than the standard deviation associated with a distribution generated by the sum of the distributions of the long dimension of the lobes ($\sigma _{LongSum}^{Holo} =1.1\,\textrm{nm}$, $\sigma _{LongSum}^{Apo}=1.6\,\textrm{nm}$).

In summary, the statistical analysis of the molecular size distribution strongly points to a length variability of the connecting region between the two lobes for both *apo*- and *holo*-transferrin molecules, which appears to allow for an extension of the molecule while retaining the lobes’ overall dimensions.

**Table 1:** Mean values and standard deviations of Gaussian fits of the size distributions in Figure 3 and comparison of the full molecule length distribution to the sum of two long lobe dimension distributions. Lobe dimensions fit well within the maximum and minimum dimensions that can be measured from the respective crystallographic models (maximum dimension *holo* = 6.5 nm and *apo* = 6.8 nm; minimum dimension *holo* = 4.1 nm and *apo* = 4.8 nm). The comparison of the experimentally measured full molecule length and the sum of the lobe dimensions indicates a broadening of the full molecule length distribution which is in agreement with the observed elongation of the experimentally observed molecules with respect to the crystallographic models, which yield the following projected full-length dimensions: 9.5 nm for *holo*- and 10.5 nm for *apo*-transferrin.

	Lobe long dimension (nm)		Lobe short dimension (nm)		Full molecule length (nm)		Sum of lobe long dim. (nm)	
	Holo	Apo	Holo	Apo	Holo	Apo	Holo	Apo
Mean value ($\mu$)	5.1	5.8	4.3	5.0	11.5	12.4	10.2	11.6
Standard deviation ($\sigma$)	0.8	1.1	0.7	0.8	2.4	2.5	1.1	1.6

## Conclusion

We have shown that low-energy electron holography imaging can map conformational differences intrinsically tied to the molecules’ biological function which manifest on a spatial scale of approximately 1 nm on the single-molecule level as demonstrated by the structural differences observed in the reconstructed images of *holo*- and *apo*-transferrin. Additionally, the single-molecule nature of the LEEH measurement allows us to draw conclusions regarding the flexibility of the region connecting the two lobes from the analysis of the molecules’ conformational space. Since many protein systems undergo conformational changes while fulfilling their biological role, the imaging of such structural differences on the single-molecule level could be of great relevance for the study of a large range of other proteins in a biological or biomedical context. Transferrin itself could be a system of interest for further investigation since it in principle can not only bind iron, but also other metal ions or small molecules^8,13,19,20^, which, along with its ability to cross the blood brain boundary, makes it a promising candidate for targeted drug delivery.

## Methods

### Low-energy electron holography

The LEEH microscope^3,4,21,22^ used to obtain the experimental protein holograms features a lens-less in-line holography geometry^23,24^, i. e. the electron source, the sample and the detector are aligned along the same optical axis. Sharp tungsten tips terminating in a few atoms are used as coherent electron sources, which field-emit low-energy electrons (30-150 eV) when in measuring distance to the SLG substrate (approximately 100-500 nm), which allows the imaging of individual protein molecules. The magnification is purely geometrical and can be adjusted by a change in tip-sample distance. The hologram is formed as the interference pattern resulting from the superposition of the wave scattered by the proteins and the unscattered reference wave. The hologram is recorded as a photograph of the fluorescent screen of a microchannel plate detector and is reconstructed numerically via a wave field propagation-based algorithm^18,25^. The propagation can be described by a Fresnel-Kirchhoff diffraction integral of the form:

1 $$\begin{aligned} U(x,y)=-\frac{\textrm{i}}{\lambda }\int _{-\infty }^{\infty }\int _{-\infty }^{\infty } H(X,Y)R(X,Y)\frac{e^{-\textrm{i}k\rho }}{\rho } \textrm{d}X\textrm{d}Y, \end{aligned}$$

where *H*(*X*, *Y*) is the measured hologram, *R*(*X*, *Y*) denotes the reference wave and $\rho =\sqrt{(X-x)^2 + (Y-y)^2 + (Z-z)^2 }$. The resulting wave field *U*(*x*, *y*) approximates the wave scattered by the object (exit wave). The absolute value of the exit wave yields the amplitude reconstructions presented in the main text, which as a first approximation can be viewed as a two-dimensional projection of the molecule along the optical axis. To simplify the numerical evaluation of the above integral, it can be rewritten as a series of Fourier transforms by employing the convolution theorem^18^.

The LEEH microscope operates in an ultrahigh vacuum (UHV) environment (base pressure of $1*10^{-10}\,\textrm{mbar}$ and under room temperature conditions. It is set up in an electromagnetically shielded box and is situated on a heavy concrete block which is decoupled from the building to minimise vibrations^22^, during the measurement, only ion getter pumps are used to pump the chamber. The tungsten tips are prepared by electrochemical etching in a 20 wt% NaOH solution and subsequent self-sputtering and annealing procedures in UHV. The measurements are conducted in constant current mode, with typical currents in the range of 10 nA, yielding electron doses in the range of $10^5$ electrons per second per Å$^2$. Both the illumination area and the emission voltage are usually stable over the course of a measurement session, i.e. over a few hours.

To create an undistorted reference wave, which is vital for LEEH imaging, an atomically clean, electron-transparent substrate is required. SLG fulfils these requirements as it is transparent to electrons in the relevant energy range and can be prepared in an ultraclean fashion^26^. Furthermore, graphene is conductive, which reduces charge-induced distortions and artefacts. In particular, a strong overlap of the electronic states of the graphene with those of the proteins indicate a critical role of the graphene in avoiding molecular charging during the electron irradiation^27^. Additionally, its weak interaction with biomolecules minimizes structural changes to the imaged proteins resulting from substrate interactions^28^.

For a detailed description of the experimental setup, the tip preparation and characterization as well as the graphene and protein sample preparation see Szilagyi^22^.

The data presented in this paper has been collected over the course of several experiments for both *holo*- and *apo*-transferrin. While not all data has been acquired using the same tip, the tip parameters have been kept as similar as possible to ensure high-quality hologram data. Each hologram is reconstructed at a range of tip-sample distances resulting in a *z*-stack of two-dimensional images. The focal distance is determined from this stack via the sharpness of the images. Because of the geometrical nature of the magnification, the determination of the focal distance also directly yields the size of the field of view for each reconstructed image. As this is done separately for each image, reconstructions obtained from holograms acquired with different tips or different imaging parameters can directly be compared.

### Sample preparation

To achieve the ultraclean sample conditions required for LEEH imaging, the proteins are deposited on SLG by native ES-IBD^17^. This not only allows a clean and controlled sample preparation, but also ascertains that the deposited molecules are chemically intact via mass spectrometry and mass selection procedures before deposition^2,3,17,29^. The spray solutions were created from lyophilized *apo*- and *holo*-transferrin (Sigma Aldrich) dissolved in 200 mM ammonium acetate and purified via buffer exchange columns. Nanoflow pulled glass capillary tips, operated at low voltages in the range of 1-1.5 kV were employed to retain a native state of the proteins during the electrospray process. The concentration of the spray solutions was in the range of 0.5-1 mg/ml. The proteins are soft-landed (kinetic energy upon landing below 5 eV per charge) on SLG in UHV. The coverage is controlled by monitoring the deposited charge, using a picoampmeter, to ensure sparse samples that allow the imaging of individual molecules.

### Native mass spectrometry

Native mass spectra (Supplementary Fig. 1) were recorded on a Q Exactive UHMR Orbitrap mass spectrometer (Thermo Fisher Scientific). Protein solutions in 200 mM ammonium acetate were prepared and sprayed as described above. General instrument conditions were as follows: Source DC offset = 21 V, S-lens RF level = 200 (300 Vp-p), transfer capillary temperature = 60°C, ion transmission settings set to “High mz” (700 Vp-p for the injection flatapole, and 900 Vp-p for the bent flatapole, transfer multipole, and collision cell), detector optimization “High mz”, injection flatapole = 5 V, interflatapole lens = 4 V, bent flatapole = 2 V, transfer multipole = 0 V, collision-cell pressure setting 7 ($N_2$), HCD CE 5. Native mass spectra confirm that the solutions used for LEEH sample preparation contained exclusively *apo*- and *holo*-transferrin and no mixtures or species with a single bound iron ion.

## Supplementary Information

Supplementary Information.

## Data Availability

The datasets used and analyzed during the current study are available from the corresponding author on reasonable request.
