# Supplementary material for: Imaging conformations of holo- and apo-transferrin on the single-molecule level by low-energy electron holography
Source: Sci Rep. 2023 Jun 23;13:10241. doi: 10.1038/s41598-023-37116-x (PMC10290138; doi:10.1038/s41598-023-37116-x)
Supplement: Supplementary file 1 — Supplementary Information. [file 41598_2023_37116_MOESM1_ESM.docx]

**Supplementary information for “Imaging conformations of *holo*- and *apo*-transferrin on the single-molecule level by low-energy electron holography”**

Hannah Ochner^1,*^, Sven Szilagyi^1^, Moritz Edte^1^, Tim K. Esser^2,a^, Stephan Rauschenbach^1,2^,
Luigi Malavolti^1,*^, and Klaus Kern^1,3^

^1^Max Planck Institute for Solid State Research, Heisenbergstr. 1, 70569 Stuttgart, Germany

^2^Department of Chemistry, University of Oxford, 12 Mansfield Road, Oxford OX1 3TA, UK

^3^Institut de Physique, École Polytechnique Fédérale de Lausanne, 1015 Lausanne, Switzerland

^a^Present address: Thermo Fisher Scientific, 1 Boundary Park, Hemel Hempstead, HP2 7GE, UK

^*^Corresponding authors:
Hannah Ochner (h.ochner@fkf.mpg.de),
Luigi Malavolti ([l.malavolti@fkf.mpg.de](mailto:l.malavolti@fkf.mpg.de))

**
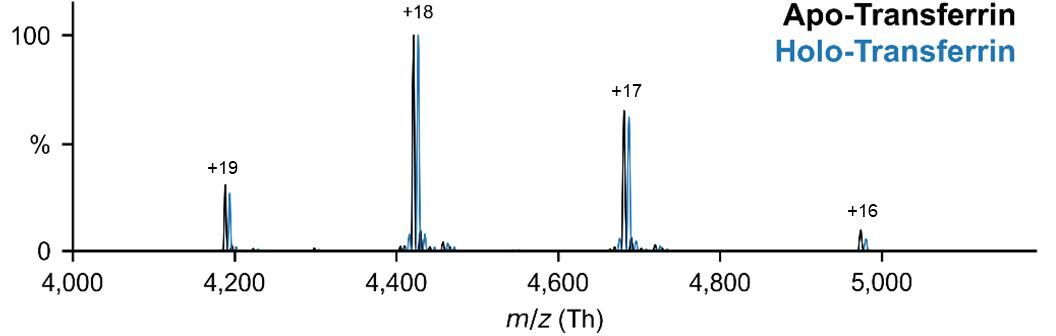
**

**Supplementary Figure 1:** Native mass spectra from apo- and holo-transferrin solutions used for LEEH sample preparation. Both spectra show the charge state distribution of the monomer with the highest intensity at charge state +18. The difference between the corresponding masses is 107 Da, which deviates by less than 5 % from the expected mass difference of 112 Da for two iron atoms. Peaks corresponding to species other than the expected zero or two iron atoms are not found. This clearly shows that the solutions contained exclusively apo- and holo-transferrin and no mixtures or species with a single bound iron ion.


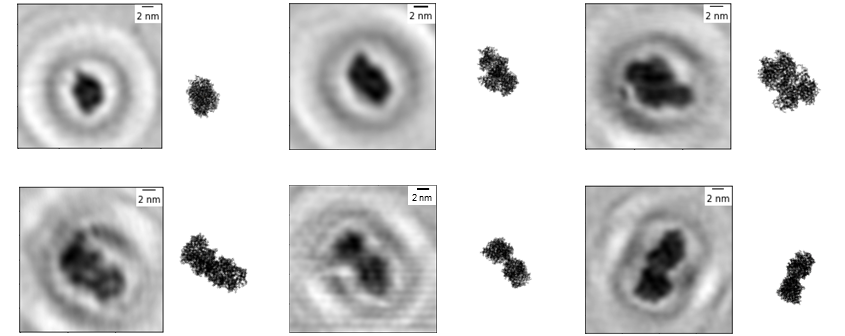


**Supplementary Figure 2**: Examples of reconstructed amplitude images of holograms of individual holo-Transferrin molecules exhibiting a range of different molecular shapes corresponding to different orientations with respect to the graphene surface. We observe a similar range of molecular shapes for apo-transferrin. While many molecules (approx. 40-65%) are observed in extended two-lobe orientations with varying degrees of lobe separation, more compact structures can also occur. A matching projection from the crystallographic model (PDB: 1JNF, shown to the right of each example) can be found for each of the experimentally observed molecules, which suggests that the molecules remain structurally intact on the length scale of the experimental resolution during the ES-IBD sample preparation and the LEEH imaging process.

**Cleft opening visibility from *apo*-transferrin PDB structure**

In order to evaluate the cleft visibility using projections obtained from the crystallographic model of *apo*‑transferrin, a dedicated PyMol plugin, named *cavity_visibility*, was developed. The code uses the apo‑transferrin *1ryx* pdb model [1] with atom coordinates defined with respect to the *XYZ* axis as reported in Supplementary Figure 3. In our model, an additional object is added inside each cleft opening: *object_1* with coordinates (36.6 Å; 94.9 Å; 3.8 Å) inside the N‑lobe cleft (green sphere in Supplementary Figure 3) and *object_2* with coordinates (19.3 Å; 42.5 Å; 23.5 Å) within the C‑lobe cleft (purple sphere in Supplementary Figure 3). These objects are used to mark the clefts’ opening and their positions have been selected to be in the proximity of the amino acids comprising the Fe binding site in the *holo*-transferrin form. After having defined a specific molecular orientation (Supplementary Figure 4a), the code creates two projections onto the *x’y’* plane: *1ryx* +*object_1* and *1ryx* +*object_2*. In these processes, each atom of the protein is projected onto the *x’y’* plane in the form of a disk of radius 3 Å, which is associated with a positive value (*p*), whereas *object_1* and *object_2* are defined as a disks with negative values (*o*) and a radius of 9 Å (r_o_), respectively. Empty spaces are assigned the value zero. The final projections are obtained by summing all atom contributions (see Supplementary Figure 4b). Since we choose $\left| o \right|<\left| p \right|$, negative values are thus present in the projections if and only if a disk associated with a cleft marking object is visible. By integrating over the negative-valued pixels and renormalizing to a 1 Å^2^ integration area, the visible cleft area (cleft visibility) expressed in Å^2^ is obtained. To define whether a cleft is visible according to the calculated cleft visibility value, we thus set a threshold of 12 Å^2^, which roughly matches our current experimental resolution. For each molecular orientation, the visibility of the C-lobe and N-lobe clefts are independently obtained and saved. The code repeats this projection process *N* times (*N* = 5x10^3^ for the data presented in main text), every time using a random *1ryx* +*object_1*+*object_2* view corresponding to a random rotation of the molecule with respect to the *x’y’* plane. The resulting cleft visibility data was used to create the graph reported in Fig. 2a (model).


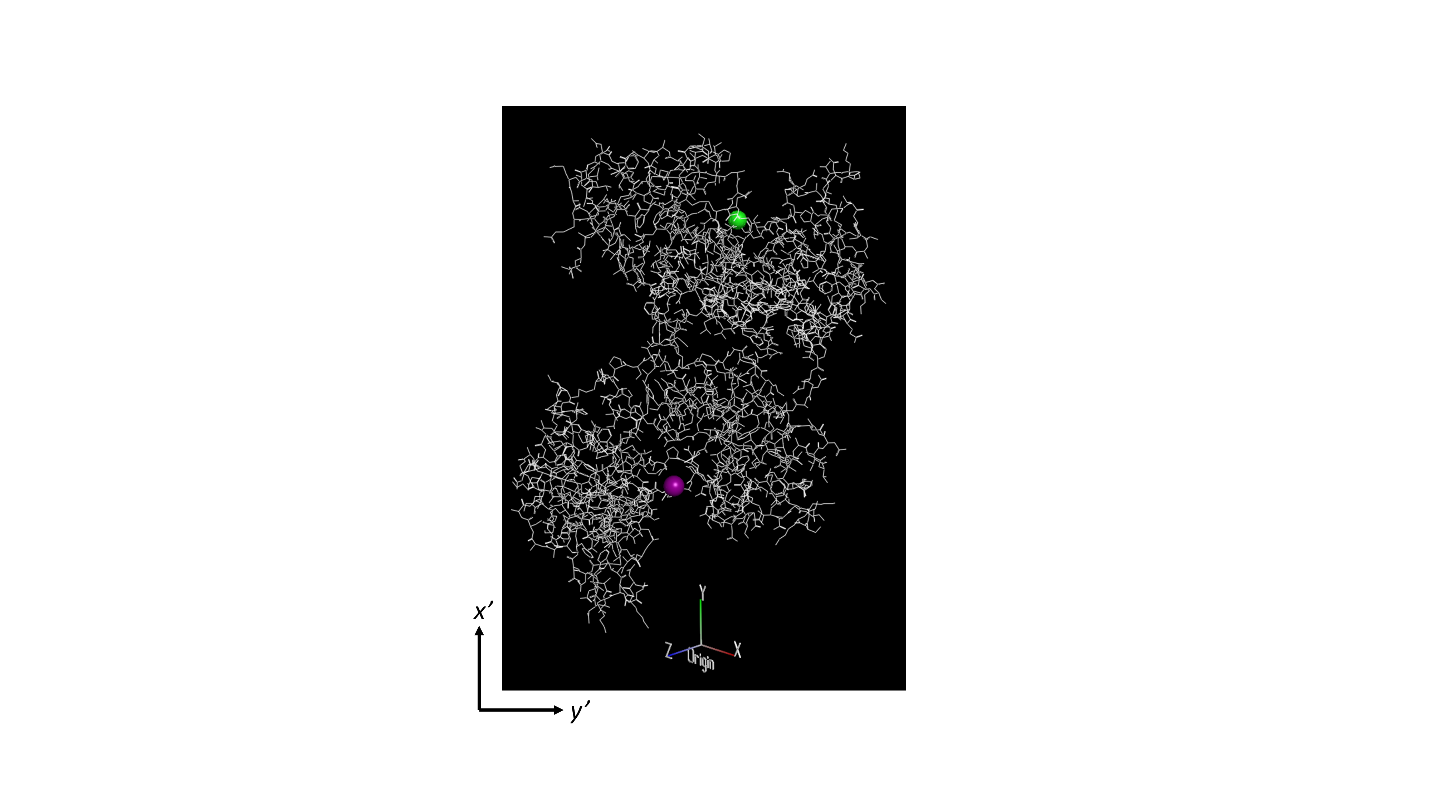


**Supplementary Figure 3:** Wire representation of the apo-transferrin model (1ryx [1]) along with object_1 in green and object_2 in purple. The atom coordinates are defined with respect to the *XYZ* axes while the *cavity_visibility* code projects them on the *x’y’* plane.

To estimate the error associated with this process, we have initially evaluated whether 5x10^3^ iterations adequately sample all the possible orientations. By successively running the code 5 times with r_o_ = 9 Å, we observe a standard deviation of the cleft visibility data of 0.25%. This uncertainty is negligible compared to the variations obtained by considering different r_o_ values. We have thus used this second method for error estimation. In particular, we have considered r_o_=12 Å, a value resulting in a cleft marking object size close to the full width of the cleft opening, for an upper limit of cleft visibility, and
r_o_ = 7 Å as the other extreme (see Supplementary Figure 4b and Supplementary Table 1).


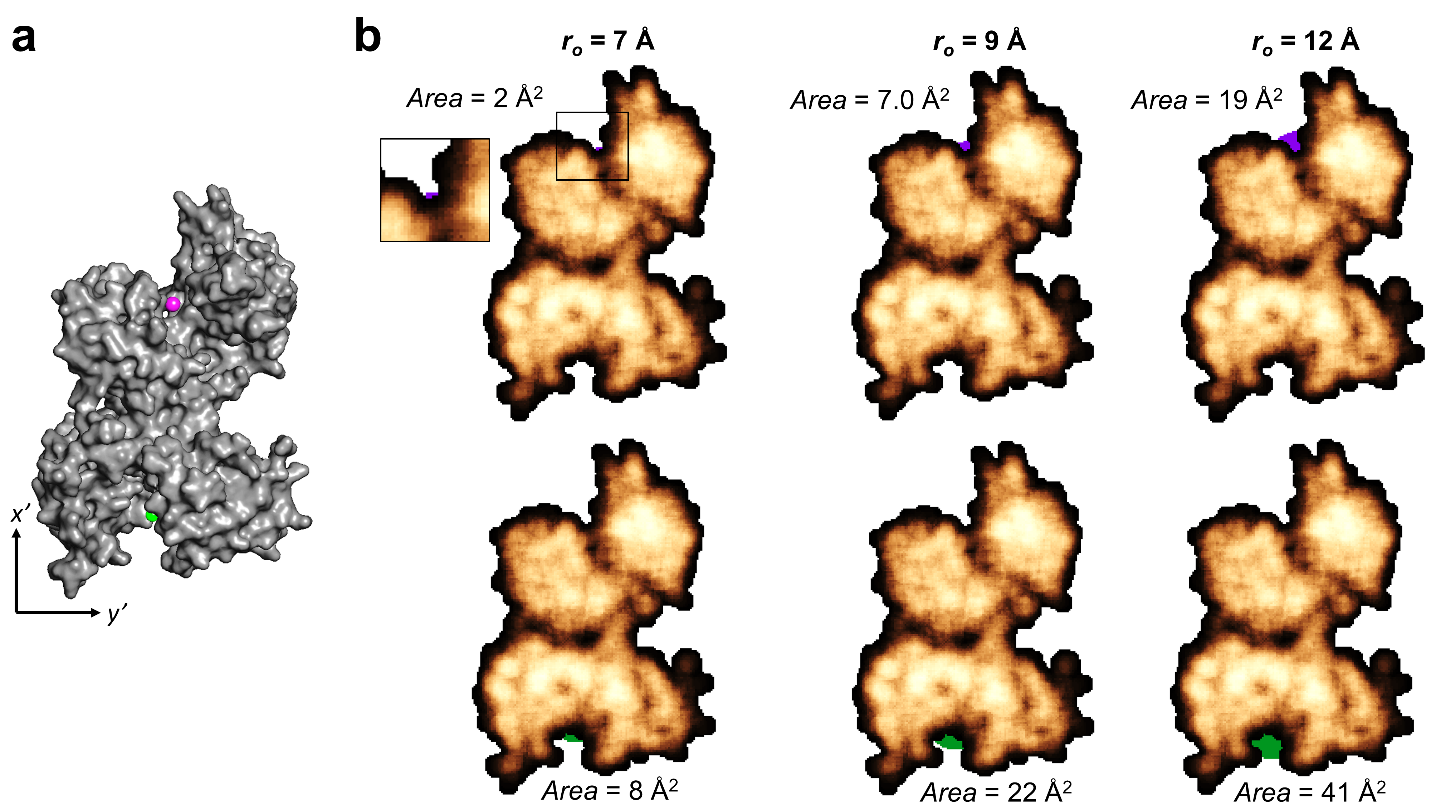


**Supplementary Figure 4:** **a** Surface representation of the apo-transferrin model (1ryx [1]) along with object_1 in green and object_2 in purple used for obtaining the projections reported in b. **b** Projections of the model presented in a using the cavity_visibility code; on top (bottom) the cavity visibility area of the C Lobe (N-lobe) is reported for three different r_o_-values: 7 Å, 9 Å and 12 Å (left to right).

| **Visible clefts** | **r_o_ = 7 Å** | **r_o_ = 9 Å** | **r_o_ = 12 Å** |
| --- | --- | --- | --- |
| **0** | 69% | 63% | 55% |
| **1** | 31% | 37% | 42% |
| **2** | 0% | 0% | 3% |

**Supplementary Table 1:** Percentage of visible clefts obtained from 5x10^3^ random projections for different r_o_-values using an area threshold of 12 Å^2^.

**References**

[1] Thakurta, P. G., Choudhury, D., Dasgupta, R. & Dattagupta, J. K. Tertiary structural changes associated with iron binding and release in hen serum transferrin: A crystallographic and spectroscopic study. *Biochem. Biophys. Res. Commun.* **316**, 1124–1131 (2004).
